# Evaluating Peripheral Vascular Injuries: Is Color Doppler Enough for Diagnosis?

**Published:** 2014-01-01

**Authors:** Mohd Lateef Wani, Mohamad Tufail Sheikh, Nayeem-ul-Hassan, Ifat Irshad, Abdul Gani Ahangar, Farooq Ahmad Ganie, Mohd Tafazul Sheikh, Shadab Nabi Wani

**Affiliations:** 1 Department of Cardiovascular and Thoracic Surgery, SheriKashmir Institute of Medical Sciences, Srinagar, India; 2 Department of Radiodiagnosis, Sheri Kashmir Institute of Medical Sciences, Srinagar, India

**Keywords:** Color Doppler, Vascular Injury, Anastomosis

## Abstract

**Background:**

Vascular injury poses a serious threat to limb and life. Thus, diagnosis should be made immediately with minimally invasive methods. Doppler is a good aid in diagnosis of vascular injury.

**Methods:**

The present prospective study was conducted on 150 patients who presented with soft signs (the signs which are suggestive but not confirmatory) of vascular injury. They were subjected to color Doppler examination before exploration. The patients with the features of vascular injury on color Doppler were subjected to exploration. On the other hand, those who had normal Doppler were subjected to CT- angiography. Then, the findings of the exploration were matched with those of color Doppler. The data were analyzed using the SPSS statistical software.

**Results:**

Out of the 150 Doppler examinations, 110 (73.33%) were reported as positive, while 40 were reported as negative for vascular injury. These were subjected to CT-angiography and seven of them had the features of vascular injury on CT-angiography. All the patients with positive Doppler or CT angiography findings were subjected to exploration. Doppler had a sensitivity of 94% and specificity of 82.5% in diagnosis of vascular injury using Binary classification test.

**Conclusions:**

Color Doppler is an easily available, reliable, and handy method of diagnosing a vascular injury. It has a very high sensitivity and specificity in diagnosis of vascular injuries.

## 1. Background

Vascular injury presents a great challenge to emergency residents because these injuries require urgent intervention to prevent loss of life or limb and also because sometimes serious vascular injury presents only with subtle or occult symptoms or signs. Such patients may present weeks or months after the initial trauma with symptoms of vascular insufficiency or embolization, pseudo aneurysm, arteriovenous fistula, etc (1-3). Although the majority of vascular injuries are caused by penetrating trauma from gunshot wounds, stabbing, and blast injury, possibility of vascular injury needs to be considered in the patients presenting with displaced long bone fractures, crush injury and prolonged immobilization in a fixed position by tight casts or bandages and various invasive procedures. Iatrogenic vascular injuries constitute about 10% of the cases in most series. Yet, their incidence is following an increasing trend due to performance of more endovascular procedures, such as angioplasty and cardiac catheterisation. Examination of the patients with features of vascular injury begins with palpation of peripheral pulses. Although the absence of pulse is an unreliable sign, it is very important and warrants further investigation rather than immediate surgery. A false positive pulse deficit may occur in shock, segmental vasospasm, compressive dressings and casts, congenital absence of pulse, and preexisting vascular diseases. On the other hand, false negative signs are found in case of strong collateral establishment. Vascular injuries are often evaluated by invasive and non-invasive tests where there is a diagnostic dilemma. Non-invasive tests include hand held Doppler, Ankle brachial index, B mode ultrasound, Duplex ultrasound, and Color Flow Doppler ultrasound. Invasive tests also include conventional angiography, CT angio, Magnetic Resonance Angiography (MRA), and Digital Subtraction Angiography (DSA). The present study aims to analyze the efficacy of color Doppler in diagnosis of vascular injury (3-5).

## 2. Materials and Methods

The present prospective study was conducted on the patients admitted from May 2006 to May 2011. On arrival, the patients were admitted in Accidental and Emergency Department and were resuscitated as per the vital status of the patient. Initial resuscitation was started by basic A, B, C (airway, breathing, and circulation). Moreover, pressure bandaging and clamping of vessel by bulldog clamp was done to achieve control over the bleeding vessel in case of obvious vascular injury. I / V fluids, e.g. crystalloids, colloids, and blood transfusion after cross matching through two wide bore canulae at available sites were given as per the requirements. Urinary catheterization was done in every patient. Associated injuries were taken due care of by the respective departments. The patients were categorized into two groups depending upon the clinical status:

Category 1 (Hard Signs): These included pain, pallor, pulselessness, parasthesias, paralysis, pulsatile bleeding, and large or expanding haematoma. A patient who shows these signs will have > 90% chance of vascular injury.

Category 2 (Soft Signs): These included a relatively diminished but palpable pulse, a non expanding haematoma, and peripheral nerve injury. Overall, 3035% of these patients will have vascular injury.

The patients whose vascular injury was obvious and those who were hemodynamically unstable were directly shifted to emergency theatre and explored. Others were subjected to vascular Color Doppler before exploration. Color Doppler was done by the radiologist on duty and the findings were noted down in the pro forma. The patients with features of vascular injury on color Doppler were subjected to exploration. On the other hand, those who had normal Doppler were subjected to CT-angiography. CT-angio is the gold standard for diagnosis of vascular injury. The findings of exploration were matched with those of color Doppler. After all, the data were statistically analyzed using the SPSS statistical software.

## 3. Results

The present prospective study was conducted on 384 patients of suspected vascular injury were studied prospectively. 234 had hard signs of vascular injury and 150 had soft signs of injury. The patients with the hard signs of injury were directly shifted to theatre. On the other hand, the remaining 150 patients underwent color Doppler examinations before exploration. Doppler was done by the radiologist on duty. Out of the 150 Doppler examinations, 110 (73.33%) were reported as positive, while 40 were reported as negative for vascular injury. These were subjected to CT angiography and seven of them showed the features of vascular injury. All the patients with positive Doppler or CT-angiography findings were subjected to exploration. On exploration, 72 patients had transected vessels, 38 had contusion with thrombosis, and 7 had lateral tear only. The patients with negative color Doppler and CT-angiography were managed conservatively. It should be mentioned that all these patients had normal vascularity. In addition, 65 patients were managed by reverse saphenous vein graft. Besides, 48 patients had end to end anastomosis and four patients underwent lateral repair only. Doppler had a sensitivity of 94% and specificity of 82.5% (95% confidence) in diagnosis of vascular injury. Moreover, binary classification test revealed its positive and negative predictive value to be 100% and 85.10%, respectively.

## 4. Discussion

The prevalence of vascular injuries is rising because of increase in the rate of invasive procedures and traumatic events. Vascular injury can pose a serious threat to limb and life if not diagnosed well in time. Delay in diagnosis can tell upon the limb salvage (1-5). Most of the time, diagnosis is obvious (hard signs of vascular injury). However, in doubtful cases, investigations such as hand held Doppler, Color Doppler, CT-angiography, and MR-angiography can aid the diagnosis (6). Most researchers have reported that around 6080% of vascular injuries can be diagnosed by proper clinical assessment (7, 8). Color Doppler is a great instrument to aid the diagnosis. Angiography is also the gold standard for diagnosis; however, because of its invasiveness and time consuming process, its role in vascular injury is limited. Most of the authors have found that arteriography has a limited role in diagnosis of vascular injury in acute setting (9-12).

Color Doppler is an easy to perform investigation. It has 95% sensitivity, 99% specificity, and 98% accuracy in assessment of peripheral vascular injuries. 100% sensitivity and specificity compared with the conventional arteriography and operative exploration by Fry and colleagues 1994 (13). Bergstein et al. (14) conducted a study entitled “Pitfalls in the use of colour flow duplex (CFD) ultrasound for screening of suspected arterial injuries in penetrating extremities” in 1992 at Medical College of Wisconsin, Milwaukee. They compared CFD with arteriography in 67 patients without obvious vascular injuries. With arteriography as the “Gold Standard”, CFD had a specificity of 99%, sensitivity of 50%, and negative and positive predictive values of 66% and 7%, respectively. With these three caveats, CFD scanning may be useful for screening the extremities with penetrating injuries thought to harbor occult arterial injuries.

Color Doppler is an easily available, reliable, and handy method of diagnosing a vascular injury. This method has a very high sensitivity and specificity in diagnosis of vascular injuries.
